# Effects of COVID-19 on cognition and brain health

**DOI:** 10.1016/j.tics.2023.08.008

**Published:** 2023-11

**Authors:** Sijia Zhao, Sofia Toniolo, Adam Hampshire, Masud Husain

**Affiliations:** 1 Department of Experimental Psychology, University of Oxford, Oxford OX2 6GG, UK; 2 Nuffield Department of Clinical Neurosciences, John Radcliffe Hospital, University of Oxford, Oxford OX3 9DU, UK; 3 Wellcome Trust Centre for Integrative Neuroimaging, Department of Experimental Psychology, University of Oxford, Oxford OX2 6AE, UK; 4 Department of Brain Sciences, Imperial College London, 926 Sir Michael Uren Hub, 86 Wood Lane, London W12 0BZ, UK

**Keywords:** long COVID, post-COVID condition, neuropsychology, neuroimaging, MRI, FDG PET

## Abstract

COVID-19 is associated with a range of neurological, cognitive, and mental health symptoms both acutely and chronically that can persist for many months after infection in people with long-COVID syndrome. Investigations of cognitive function and neuroimaging have begun to elucidate the nature of some of these symptoms. They reveal that, although cognitive deficits may be related to brain imaging abnormalities in some people, symptoms can also occur in the absence of objective cognitive deficits or neuroimaging changes. Furthermore, cognitive impairment may be detected even in asymptomatic individuals. We consider the evidence regarding symptoms, cognitive deficits, and neuroimaging, as well as their possible underlying mechanisms.

## Highlights

Even months after recovery from COVID-19 infection, some people continue to experience neurological, psychiatric, and cognitive effects.
Recent extensive research using electronic health records, cognitive testing, and neuroimaging has started to clarify the nature of some of these symptoms.
COVID-19 offers an unprecedented opportunity to follow individuals from acute infection through to the emergence of post-viral illness on a broad scale.

## Brain health and symptoms associated with COVID-19

Early in the coronavirus disease 2019 (COVID-19) pandemic caused by severe acute respiratory syndrome coronavirus 2 (SARS-CoV-2), it was appreciated that there could be significant effects of the infection on the brain [1]. Neurological syndromes were observed most commonly in hospitalised patients, including delirium, encephalopathy, and stroke [2., 3., 4.]. An analysis of electronic health records of >236 000 people who had been infected with COVID-19 showed that non-hospitalised patients also incurred a significantly higher likelihood of developing psychiatric conditions, including altered mood, anxiety, insomnia, and psychosis [5].

The estimated incidence of receiving either a neurological or psychiatric diagnosis for the first time was 11.5% in the first 6 months following infection, and this doubled among patients with intensive care unit (ICU) admission [5]. Importantly, these figures cannot simply be explained by systemic effects associated with any respiratory tract infection because the rates were significantly higher compared to patients with influenza [5,6]. However, some studies found no difference between COVID-19 and other respiratory tract infections with comparable disease acute illness severity [7,8]. Some of the elevated risks appear to be transient. After 1–3 months it has been reported that diagnostic rates for stroke, insomnia, and mood and anxiety disorders gradually return to levels comparable to those of patients with other respiratory tract infections [9]. Nevertheless, following the observation of the acute impact of COVID-19 infection on the brain, it soon became clear that there were also symptoms in the chronic phase [10]. Two years after infection, the risks of cognitive symptoms, dementia, and psychotic disorders were still significantly elevated [9].

The prevalence and persistence of self-reported cognitive symptoms after COVID-19 have been extensively reported, but the variability in estimates between studies is very large [11., 12., 13., 14.]. In a longitudinal study on 766 infected individuals, 36% reported cognitive symptoms in the first 3 months following infection, and those with cognitive symptoms in the first month were twice as likely to report chronic symptoms at 3 months compared to those without such symptoms [15]. According to a recent meta-analysis of online surveys, one in five individuals who contracted COVID >3 months ago still continue to experience some degree of 'brain fog', including attention and memory problems [16].

These self-reported cognitive complaints are amongst the most common symptoms of the post-COVID condition (PCC), also known as 'long COVID' [17]. According to the World Health Organization clinical case definition, PCC comprises a constellation of symptoms (e.g., cognitive dysfunction, fatigue, shortness of breath) that are still present 3 months after coronavirus infection, have lasted at least 2 months, affect daily functioning, and cannot be explained by another cause [18]. Given the heterogeneity of PCC symptoms, it has been proposed that PCC might not be a unified condition and instead reflects a group of distinct phenotypes [19., 20., 21., 22.].

Of symptomatic COVID-19 patients, 2.2% experienced chronic cognitive symptoms 3 months after infection, according to a meta-analysis of 1.2 million cases [22]. Recent estimates suggest that 1.7 million UK adults (2.7% of the population) had self-reported PCC in March 2023, and 69% had symptoms for >1 year [23]. However, the prevalence of chronic cognitive symptoms could be overestimated in many studies. One study found the prevalence of cognitive symptoms was the same (70%) regardless of whether individuals had been infected or not [24], highlighting potential difficulties in interpreting the prevalence of symptoms. It is therefore crucial to consider data from studies that have objectively measured cognitive function rather than relying on self-report.

## Cognitive function in the acute phase

Global, clinical cognitive screening tests were commonly used in the acute phase, namely within 3 months of infection [25., 26., 27.]. For example, a comprehensive examination of 49 largely non-hospitalised COVID-19 patients with an average age of 60 years showed lower scores on mini mental state examination (MMSE) compared to age-matched controls, and 53% were significantly impaired in at least one cognitive domain (attention and executive, memory, or visuospatial) using a more extensive neuropsychological battery 2–3 months after infection [28]. This accords with a meta-analysis of 24 studies, all using cognitive screening tests, which reported that 52% of patients overall demonstrated acute deficits, and those aged over 59 years fared worse [26]. However, most of the publications in this analysis focused on hospitalised patients, namely those with more severe manifestations. Thus, there is likely to be a bias in patient selection.

One might argue that surviving any critical illness can result in long-lasting cognitive impairment [29], and this reported effect might therefore not be specific for SARS-CoV-2 infection. To address this question, a case–control matched study [30] found that, compared to 60 ICU status-matched uninfected patients, 85 COVID patients had significantly lower Montreal cognitive assessment (MoCA) scores at 6 months post-discharge, and more COVID-19 patients scored below the cut-off for mild cognitive impairment.

Some important limitations of conventional clinical screening tools are that they are relatively crude measures that may not be sufficiently sensitive to detect mild cognitive deficits. They also do not lend themselves to mass testing or longitudinal research owing to lack of different versions of tests (to mitigate against practice effects) and the need for face-to-face testing. During the pandemic there was great interest in digital cognitive testing in which people complete computerised tests remotely. Hampshire *et al.* [31] reported data from 81 337 people who performed an online suite of cognitive tests. Of these, 12 689 people were verified (*n* = 518) or suspected to have COVID-19, and the majority had been infected 2 months previously. The authors found cognitive impairment scaled with acute COVID-19 respiratory severity: hospitalised patients scored 0.25–0.45 standard deviations below the mean of the uninfected healthy group, whereas non-hospitalised patients showed a lesser but still significant reduction in global cognitive score, and this could not be attributed to premorbid intelligence or level of anxiety and depression symptoms in the weeks before testing. Regardless of acute symptom severity, the most affected domains were semantic analogical reasoning (e.g., 'leaf is to branch' as 'finger is to arm') and multi-stage planning (measured by the Tower of London test). Box 1 overviews deficits in cognitive functions, such as executive function and memory, in the acute phase of COVID-19.

Box 1 **Executive dysfunction and memory deficit in the acute phase**

Executive dysfunction is a commonly reported effect in the acute stage, especially in patients over 60 years of age with moderate to severe acute symptoms [33,74,124]. However, it has been difficult to determine the frequency and magnitude of acute executive dysfunction because different tasks and measures have been deployed. The most common test used is the trail-making task (TMT). Impairment on this task has been frequently reported in the acute phase, especially among people over 50 years of age who had moderate to severe respiratory symptoms [53,125] or cognitive symptoms [126], but this is much less common in young non-hospitalised cases [127,128]. However, pre-existing illnesses, such as diabetes, may alter the prevalence of cognitive symptoms [129]. More advanced executive functions, such as multi-stage planning, may also be impaired in some individuals [31].

Infected people have also consistently demonstrated impairment in memory using standard screening tools as well as specific memory tasks such as free recall of wordlists and recall of drawing complex figures [25,28,79,130,131].

Alt-text: Box 1

Other investigations also report that the severity of cognitive impairment depends on the level of medical assistance needed during the acute SARS-CoV-2 infection [31,32], especially if intensive care and oxygen support were required [28]. However, the length of hospitalisation has been shown not to be related to the acute cognitive deficit [33,34]. The association with acute symptom severity has also been reported to be particularly strong in executive dysfunction; one study objectively measured this using the number symbol coding task and found a correlation with an index of acute respiratory distress [28].

## Cognitive function in the chronic phase

Some COVID-19 survivors may have attention, executive function, and memory deficits for >3 months [28,35., 36., 37., 38., 39., 40., 41., 42., 43., 44., 45.], and potentially up to 2 years if they have ongoing symptoms [46]. The prevalence of chronic cognitive impairment remains unclear among the infected population [16,47., 48., 49.]. Importantly, despite the fact that cognitive functions are measured independently, cognitive impairment in multiple domains is more common than in a single domain [28,38,50].

Overall, the objective testing data indicate that, for the majority of mild-to-moderately infected individuals, recovery occurs within the first year after infection (Box 2 for the persistence of chronic cognitive impairment); however, some may experience some degree of cognitive deficit beyond 1 year, similar to the trajectory of self-reported PCC symptoms [51]. The presence of ongoing symptoms may also be a key factor: a digital testing study that tracked 101 self-reported PCC patients for 9 months found no sign of recovery in cognition over this time, and cognitive impairment was still evident even 2 years after infection [46].

Box 2 **How long does chronic cognitive impairment persist after COVID-19?**

Currently, outcomes differ considerably depending on the population assessed and the cognitive tests used, but in general, 1 year after infection, the majority of mild-to-moderately affected individuals would have a normal global score on cognitive screening tests. For instance, 9 months after infection, 443 primarily non-hospitalised individuals had normal mini-mental state examination (MMSE) scores [132], and 4 months after infection, 98% of 120 healthcare workers had a normal MMSE score [55]. Individuals older than 60 years (*n* = 1178) also showed normal global cognition compared to their uninfected spouses (*n* = 438) 6 months post-infection, measured through telephone interview of cognitive status (TICS) [133]. In a prospective study of 78 middle-aged residents in a rural village in South America with a pre-pandemic baseline, Montreal cognitive assessment (MoCA) scores dropped considerably 6 months after SARS-CoV-2 infection but recovered at 18 months [134]. Similarly, 53 young adults in their 30s who were infected >9 months ago performed normally on demanding computerised attention and memory tasks [36]. However, recovery can take >12 months, as shown in one online memory study with >1700 infected adults [37].

Alt-text: Box 2

### Vigilance and sustained attention

**Figure 1:**
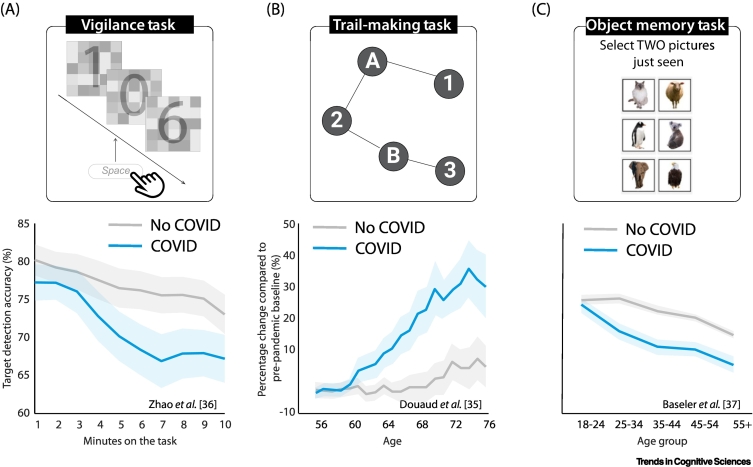
Examples of paradigms used in the literature to study the cognitive deficits following COVID-19. (A) Vigilance task designed to assess performance decrement during sustained visual attention. A single digit was presented for 50 ms every second and participants were instructed to press spacebar when seeing '0'. Young COVID survivors without ongoing symptoms (*n* = 53, mean age = 28, mean time from infections = 5 months) showed a significantly larger decrement in accuracy of detecting the target after 3 min compared to uninfected controls (*n* = 83, mean age = 29). Adapted from [36]. (B) The trail-making task (TMT) part B requires participants to connect circles in a specified order as quickly as possible, switching between numbers and letters. Completion time provides a measure of processing speed and cognitive flexibility. A total of 800 participants aged >50 years completed this task before and after the breakout of COVID-19. Those who had contracted COVID-19 (*n* = 401, mean time from infection = 5 months) required a significantly longer time to complete this task compared to uninfected controls. This suggests a deficit in executive function that becomes worse with greater age. Adapted from [35]. (C) A total of 1706 participants who had COVID-19 1–17 months previously and 3722 uninfected controls were asked to perform an object memory task. People >25 years of age showed worse performance on this test compared to their age-matched uninfected controls. Adapted from [37]. In all plots, the shaded area shows 1 standard error.

In one study on 73 diagnosed PCC patients, ~50% showed significant impairment in detecting targets on a simple visual task [41]. This is consistent with the fact that concentration difficulty is among the most reported post-COVID cognitive symptoms [23,52]. However, this attentional deficit is not only limited to people with ongoing cognitive symptoms but is also present in young individuals without subjective complaints [36]. An online testing study [36] showed that 53 young individuals who had COVID-19 5 months ago and reported no cognitive symptoms scored significantly worse on a vigilance test (Figure 1A). After only 3 min, the COVID-19 group showed a decrease in their ability to sustain attention to detect infrequent targets. The result was not due to a deficit in target detection because in the same study the COVID-19 group performed normally in the first 3 min of this attention task and also on tasks that focused on other forms of attention [36].

### Executive functions

One study [49] found that 27% of 200 hospitalised patients who had recovered from severe COVID-19 still exhibited impairment in the trail-making task (TMT) at 7 months, consistent with other evidence [40,53]. In another study, among young patients who had ongoing symptoms for ~1 year, 15% showed significant impairment in the TMT, but this effect was on average smaller than in age-matched chronic fatigue syndrome (CFS) patients [41]. This effect also seems to be age-related [35]. A very well-controlled longitudinal study in the UK Biobank compared TMT performance in 800 people to their pre-pandemic performance [35]. Individuals who contracted COVID-19 but recovered from acute symptoms took significantly longer to complete the task compared to uninfected controls. This difference persisted even after all hospitalised participants were excluded, and was especially noticeable on the TMT-B in people aged >60 years (Figure 1B). However, the same individuals performed normally on a number-coding task which requires participants to match symbols to digits based on a key provided, demonstrating some specificity of the TMT finding. Investigations of younger people – regardless of acute symptom severity – have not found any chronic effects on TMT-B performance [54] or on other executive functions such as cognitive flexibility (Wisconsin card sorting test [43]), multi-stage planning (Tower of London) [36,40,55]), and visuospatial mental rotation tasks [36,43]. In addition, working memory is also found to be affected in an online study of 1706 infected people, which also showed a strong association with age [37] (Figure 1C).

### Episodic memory

Episodic memory appears to be negatively impacted, irrespective of the types of stimuli to be remembered (words or pictures) [27,28,36,42., 43., 44., 45.,^56^,^57^]. These deficits might be observed even in relatively young and mild cases without ongoing symptoms [36]. For example, in a computerised memory task with simple line drawings of objects, 36 people in their 30s who had no ongoing symptoms performed normally on the immediate recognition test, but 30 min later they forgot more information compared to the uninfected controls [36]. The error was caused by a loss of memory details (e.g., remembering that an object in the study set was a strawberry, but forgetting its exact appearance or orientation). Despite the small sample size, a strong correlation between this episodic memory deficit and time since the onset of COVID-19 acute symptoms was found in this young group [36].

### Relation of symptoms to objective cognitive impairments

The presence of ongoing cognitive symptoms has been shown to be a strong predictor of objective cognitive deficits [37,43,46,58]. Based on a recent study, up to 2 years after infection, a moderate cognitive deficit was still detectable in people experiencing PCC (*n* = 1768, median age = 58), but not in those with perceived full recovery [46]. However, this association was absent in a slightly younger sample (*n* = 319, mean age = 49) [39]. Further, people without cognitive symptoms could also show a mild deficit months after infection [36]. Risk factors for cognitive impairments are overviewed in Box 3.

Box 3 **Risk factors for chronic cognitive impairment**

What are the risk factors for delayed recovery or even progressive cognitive decline?

**Severity of acute symptoms**

Acute COVID-19 severity is the strongest risk factor for progressive cognitive decline over 1 year, followed by hypertension, coronary heart disease, and chronic obstructive pulmonary disease, according to a longitudinal study of 1458 infected patients older than 60 years [133]. As in the acute stage, the early chronic cognitive deficit shows a relationship with acute COVID-19 severity [36,38,44,45,49,58,135]. The cognitive deficit after 9 months, however, is typically unrelated to acute COVID-19 severity [37,43], but this might vary in different age groups [133].

**Acute symptoms**

Regarding specific symptoms, intriguingly, individuals with dysgeusia (altered taste) and hyposmia during the acute stage had less recovery of memory functions after infection compared to those without these symptoms [28,45].

**Age**

Age also plays a role in chronic cognitive impairment after COVID-19 [35,37]. However, the relationship between age and cognitive deficits in COVID-19 is far from linear because some evidence points towards worse performance in young and middle-aged adults compared to older adults (50–64 years) [37,136]. Currently, we cannot exclude the possibility that different mechanisms might underlie cognitive performance in different age groups.

The literature on the impact of COVID-19 on cognition in children and adolescents is less extensive, likely due to a combination of reduced detection rates of SARS-CoV-2, lower prevalence of cognitive complaints, and difficulties in performing objective cognitive testing [137,138]. The incidence rate for cognitive symptoms at 6 months after infection (*n* = 185 748) was ~1% in this population, compared to 6% in persons >65 years (*n* = 242 101) [6]. Although potentially reassuring, a lack of cognitive symptoms does not always indicate normal cognitive function, and this therefore requires further investigation.

Older adults (>65 years) are at higher risk of being given a diagnosis of dementia 2 years after the initial infection compared to younger adults [9]. However, this effect may also be partially explained by the unmasking of pre-existing (undiagnosed) neurodegenerative illnesses. Hospitalisation with evidence of acute infection has long been known to be associated with higher rates of dementia diagnosis, beyond SARS-CoV-2 infection [139].

**Pre-existing dementia**

People with pre-existing dementia have a higher likelihood of experiencing severe COVID-19 (indexed by increased rates of hospitalisation, ICU admission, or death) [140., 141., 142.]. A study on 61.9 million adults using electronic health records in the USA reported that patients with dementia had a twofold increased risk of SARS-CoV-2 infection [141], and a 5.2-fold higher mortality rate [142] compared to patients without dementia. These suggest a bidirectional effect of neurodegenerative disease and SARS-CoV-2 infection on health outcomes [87].

**SARS-CoV-2 variants**

Preliminary evidence suggests that the specific variant of SARS-CoV-2 may also be an important risk factor. Self-reported post-COVID condition (PCC) symptoms are more common in individuals infected in the pre-omicron variant era [19,46]. Different variants (wild-type, alpha, delta) might be associated with distinct PCC symptom clusters (*n* = 9804) [143]. Noticeably, brain fog became more prevalent after infection with the delta variant than with the alpha variant [143]. However, evidence from objective testing remains limited [46].

Alt-text: Box 3

### Impact of vaccination

Numerous studies have now demonstrated that vaccinated people have lower rates of chronic cognitive symptoms than unvaccinated individuals who became infected [59., 60., 61.] (reviewed in [62]), especially if they were older [63]. However, to the best of our knowledge, no published research has made use of objective assessment, and therefore the impact of vaccines on the risk of developing new or changing existing chronic cognitive deficits in COVID-19-infected individuals remains unknown.

## Mental health and relationship to cognition

**Figure 2:**
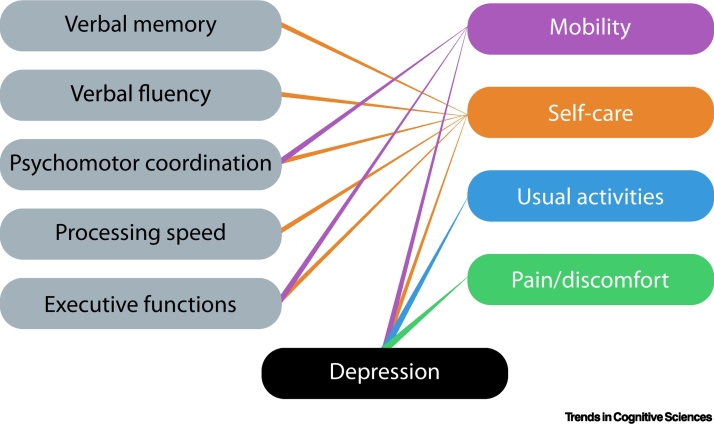
Effects of cognitive functions and depression on quality of life. Impairment on several cognitive domains (left column) had an impact on different aspects of quality of life (right column), as did the presence of depression. Adapted from [50].

Sleep disturbance, anxiety, and mood disorders can have a major impact on cognitive complaints in people [64]. COVID-19 has been linked to a high prevalence of all these symptoms [6,9,55,58,65]. However, the severity of these mental health symptoms has not consistently been shown to correlate with the degree of acute or chronic post-COVID cognitive deficits in studies with objective testing [31,36,39,58]. One study suggests that, compared to acute severity or presence of comorbidities, the presence of depression was the factor which had the greatest influence on cognitive performance [50]. Further, cognitive function and depression both had independent negative impacts on different aspects of self-reported quality of life, but in addition they also interacted significantly to influence these important metrics of PCC (Figure 2). In another study of patients attending a dedicated PCC clinic on average 5 months after infection (22% previously hospitalised), the severity of depressive symptoms was significantly associated with the degree of cognitive impairment, and this was driven mainly by verbal fluency, attention, and delayed recall deficits [56]. Sleep concerns specifically predicted memory disturbance, whereas anxiety appeared to have no significant impact on cognitive performance [56], although a modest effect of anxiety on cognition has been reported in a smaller sample [40].

Despite these associations, the nature of such observational studies means that it is difficult to establish causal, directional relationships between depression and cognitive function. Further, there is evidence that post-acute COVID-19 cognitive impairment can occur regardless of mental health symptoms in all acute severity groups [34,36,66]. This is consistent with findings from electronic health records: mental health symptoms such as anxiety and depression returned to normal within 2 months, but cognitive symptoms increased for at least 2 years [6]. Clearly, depression may play a contributory role in impaired cognitive performance, but it is also possible that post-COVID cognitive deficits and/or other mechanisms (discussed below) may induce depression. Similar issues have also been rehearsed previously in the context of CFS where the evidence does not point to a clear conclusion (discussed in [67]).

## Neuroimaging

Understanding the acute and long-term effect of COVID-19 on brain structure might provide some insights into cognitive symptoms. We focus on evidence from the three most common diagnostic methods used in the current post-COVID literature: magnetic resonance imaging (MRI), fluorodeoxyglucose positron emission tomography (FDG-PET), and electroencephalography (EEG).

### MRI

Acutely, white matter abnormalities have frequently been reported in COVID-19 patients, potentially secondary to micro- and macrovascular insults, infectious, inflammatory, or autoimmune causes [68., 69., 70., 71.]. However, even in COVID-19 patients admitted to hospital with acute neurological symptoms, MRI is normal in the majority [72]. In one study, diffusion MRI in 20 hospitalised patients revealed a reduction of intra- and extra-axonal volumes and a significant increase in the free water fraction, suggestive of vasogenic oedema, and this was highest in frontoparietal regions [73]. This type of oedema is often associated with increased permeability of the blood–brain barrier but is reversible. Another study on 58 hospitalised COVID-19 patients found higher signal on susceptibility weighted imaging sequences in the thalamus and increased mean diffusivity in both the thalamic radiation and sagittal stratum 2–3 months after disease onset [74].

**Figure 3:**
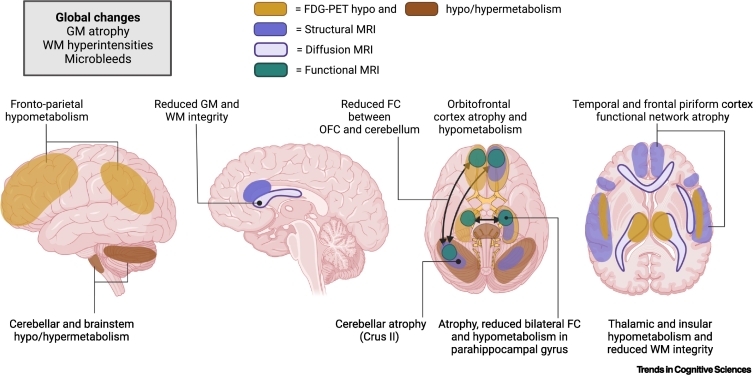
Neuroimaging findings. Several different brain regions have been reported to be affected by COVID-19 across several different modalities in some individuals. The brain regions depicted here represent those most commonly reported. On fluorodeoxyglucose positron emission tomography (FDG-PET), hypometabolism has been found in frontoparietal regions as well as in other regions including the insula, parahippocampal gyrus, and thalamus. Hyper- and hypometabolism have been described in the cerebellum and brainstem. On magnetic resonance imaging (MRI), atrophy in several brain regions connected to the temporal and piriform cortex, including the orbitofrontal cortex (OFC), parahippocampal gyrus, and cerebellar crus II, has been reported. Reduced functional connectivity (FC) between the OFC and the cerebellum, and between left and right parahippocampal gyrus, has also been described. Reduced white matter (WM) integrity has been reported in several areas, including the anterior cingulate cortex (ACC), particularly in patients admitted to an intensive care unit (ICU), as well as in the thalamic radiation. Abbreviation: GM, grey matter; Figure drawn with BioRender (biorender.com).

Chronically, the best evidence for structural changes in the grey matter in patients with COVID-19 comes from a large longitudinal UK Biobank study (Figure 3) [35]. This examined 401 people who had already had a baseline scan before the pandemic, and compared them to 384 uninfected controls. This study revealed reduced cortical thickness in brain areas functionally correlated to primary olfactory cortex such as the left parahippocampal gyrus, bilateral orbitofrontal cortex (OFC), anterior cingulate cortex (ACC), temporal pole, insula, and supramarginal gyrus in those who had COVID-19 [75]. That study also showed increased mean diffusivity changes in OFC, ACC, insula, and amygdala 5 months after infection [35]. One year after infection, one small investigation (*n* = 22) found that COVID-19 patients had diffusion abnormalities in the genu of the corpus callosum, corona radiata, and superior longitudinal fasciculus, and the corpus callosum was especially affected in people who had been admitted to ICU [76]. One study on 84 COVID-19 patients with PCC at 1 year showed reduced hippocampal volumes, particularly in hospitalised patients, and reported an association between hippocampal head volumes and cognitive performance across multiple tests [77].

An association between cognitive impairment and brain imaging has been found across different modalities. In a task-related functional MRI study, PCC patients showed greater brain activation overall when performing a demanding *n*-back memory task, and this increase in brain activation predicted the severity of the cognitive symptoms in the patients [77]. A recent multimodal MRI study on 86 PCC patients who on average were infected 11 months earlier (33% previously hospitalised) found reduced resting state functional connectivity between the left and right parahippocampal regions, as well as between bilateral OFC and the cerebellum, and functional connectivity changes in OFC were associated with memory performance [75]. Cognitive deficits including in executive functions were also found to be associated with grey matter atrophy in these areas [35,75] and white matter changes [28,71,73]. Besides single brain areas, the impact of multiple white matter hyperintensities observed in some COVID-19 patients can also contribute to their cognitive deficits in executive functions, processing speed, and memory [28]. Moreover, mental health could also be related to abnormal imaging findings in these patients. An investigation of 42 patients who were hospitalised with COVID reported that, at 3 months, severity of depression was correlated with atrophy in ACC, whereas post-traumatic symptoms were related to ACC and insular volumes; both symptoms were associated with white matter microstructural damage in several tracts and with inflammatory markers in blood [78]. These neuroimaging findings echo the aforementioned complex associations between mental health symptoms and cognition.

### FDG-PET

A pattern of frontoparietal hypometabolism on FDG-PET has consistently been reported in COVID-19 patients presenting with acute neurological symptoms [79., 80., 81., 82., 83.]. Cross-sectional data in the chronic phase as well as longitudinal analysis show that this tends to resolve over time, and there was minimal or absent residual hypometabolism after ~6 months [54,81., 82., 83.]. Other areas that have been found to be involved include the thalamus, insula, and medial temporal lobe including the parahippocampal gyrus [84., 85., 86.]. The cerebellum, pons, and brainstem have shown either hyper- [79., 80., 81., 82.] or hypometabolism [85,86]. Children have been found to show a similar pattern of hypometabolism to adults [86]. The prominent involvement of a frontoparietal network in FDG-PET imaging would be consistent with impairments in attention and executive function reported in these patients [87,88].

Overall, the findings from both MRI and FDG-PET show there can be an association between imaging abnormalities and cognitive function [28,35,73,75,79]. The true prevalence of brain imaging abnormalities in people with previous COVID-19 infection is unknown, but it remains the case that brain imaging is normal in most people with available brain imaging data with past COVID-19 infection, including PCC.

### EEG

Individuals with normal MRI scans may still have aberrant cortical activity [28,89]. EEG abnormalities, including generalised slowing and epileptiform discharges, particularly in the frontal region, are common in individuals after SARS-CoV-2 infection [89., 90., 91.]. According to two meta-analyses on 617 and 308 patients conducted early in the pandemic, COVID-19 patients can exhibit abnormal background activity in up to 96.1% of cases [91], and ~68.6% have diffuse slowing [90], whereas epileptiform discharges were less common (20.3–22.3%). Importantly, electrographic seizures, unlike clinical seizures, in COVID-19 patients have been found to be significantly associated with mortality in one study [92]. Although EEG abnormalities have been documented in patients without pre-existing neurological conditions [89,92], epileptiform discharges are increased in patients with pre-existing epilepsy [90].

COVID-19-related encephalopathy is often the major indication for performing EEG, and is associated with higher rates of abnormal findings, and frontal EEG abnormalities have been proposed as a marker of COVID-19-related encephalopathy [89., 90., 91.]. Some studies found no link between EEG abnormalities and cognitive dysfunction [89], whereas others found an association with performance during tests measuring frontal functions such as the frontal assessment battery and the TMT [28]. However, one longitudinal investigation at 10 months reported that these changes normalised over time [28].

## Possible mechanisms

Several different mechanisms, by no means mutually exclusive, have been hypothesised to account for COVID-associated cognitive impairment and changes in brain health, both in the acute and chronic phases of the disease.

### Cerebrovascular factors

MRI scans on COVID-19 patients who showed cognitive deficits or neurological symptoms indicated a high prevalence of cerebrovascular abnormalities, including ischaemic strokes, intracranial haemorrhage, and cerebral microbleeds, which could contribute to cognitive deficits [68,69,72,93., 94., 95., 96., 97., 98., 99.]. Damage to the blood vessel endothelium in acute COVID-19 infection is associated with a prothrombotic state which results in micro- and macro-thrombus formation [100]. Worse clinical outcomes are reported in patients with pre-existing endothelial dysfunction, such as patients with systemic hypertension, diabetes, or obesity [101]. The initial trigger could be direct viral invasion of endothelial cells or indirect inflammatory effects. The exposure of the subendothelium triggers the coagulation cascade and subsequent platelet activation, leading to thrombus formation, which ultimately leads to hypoxia and cell damage [102]. The presence of hypoxic–ischemic lesions has been found to correlate with neurological manifestations of COVID-19 in a large meta-analysis of 45 autopsy studies [103]. This procoagulatory state then activates increased production of proinflammatory cytokines including interleukin 6 (IL-6), IL-1, IL-10, and tumour necrosis factor α (TNF-α). This contributes to disruption of the blood–brain barrier, which in turn increases migration of monocytes from outside the brain and reinforces a proinflammatory state [104]. Age has been found to be the single most important factor in neuropathology associated with hypoxic and ischemic lesions and reactive neuroinflammation [103].

### Dysregulated autoimmunity and neuroinflammation

SARS-CoV-2 infection triggers the release of immune mediators in the blood which can manifest as a 'cytokine storm' of elevated levels of proinflammatory cytokines. Cytokine levels correlate positively with acute COVID-19 systemic severity [105., 106., 107.], and might contribute to neurological complications such as encephalopathy [108]. High levels of cytokines in the blood can also increase blood–brain barrier permeability, leading to increased migration of inflammatory cells into the brain [104]. Neuroinflammation has been consistently reported in animal and human studies at autopsy [103,109]. Heightened neuroinflammation in the hippocampus has also been found to be linked to reduced hippocampal neurogenesis in mouse and hamster models of COVID-19, and also in human samples [109,110]. Recently, a study using translocator protein (TSPO) PET reported higher distribution volume (TSPO V_T_, an index of inflammatory change) in 20 patients with depressive and cognitive symptoms after recovering from mild or moderate COVID-19 compared to 20 uninfected controls, and this was particularly notable in the dorsal putamen and ventral striatum [111]. Higher levels of TSPO V_T_ in the dorsal putamen were strongly associated with slower finger-tapping speed [111].

However, increased neuroinflammation is also found in mice and humans after mild influenza, and most cytokines normalise over time in animal models [109,110]. Reactive microglia and astroglial cells together with inflammatory infiltrates represent the most common findings and are found in 44–52% of COVID-19 cases at autopsy, and they are present regardless of detection of viral RNA, pointing towards a general inflammatory response rather than a consequence of the virus *per se* [103]. Only inflammatory infiltrates seem to be associated with rates of neurological symptoms [103]. The neuroinflammatory marker glial acid fibrillary protein (GFAP) has been found to be increased in the acute phase of COVID-19 [112,113] but, according to one study on 175 COVID-19 patients and 45 patients with influenza, levels return to their normal baseline 3 months after infection, and importantly are not different compared to samples with influenza [113]. Another study on 100 COVID-19 patients at 6 months confirmed that GFAP levels as well as initial increases in neurofilament light chain (NfL) tended to normalise over time [114]. However, one cross-sectional study on 84 patients with PCC symptoms at 1 year showed an inverse relationship between inflammatory cytokines such as CCL11 and time after COVID-19 infection, whereas other markers such as GFAP and myelin oligodendrocyte glycoprotein (MOG) did now show such an association [77]. In the same study, increased levels of GFAP and MOG were associated with higher volumes of several hippocampal subfields, unlike CCL11 and NfL which showed the opposite relationship. Therefore, although there is strong evidence to suggest that COVID-19 in the acute phase is related to neuroinflammation, more longitudinal data across different modalities will be necessary to determine whether these effects are long-standing and whether having chronic PCC symptoms affects the return to baseline of these biomarkers.

### Direct viral invasion

SARS-CoV-2 uses its spike protein to bind to angiotensin-converting enzyme (ACE) 2 receptors, which are found on the surface of cells in many organs including the lungs, heart, blood vessels, kidneys, liver, and gastrointestinal tract [115]. Although ACE2 receptors are also present on endothelial cells in the brain, there is no substantial evidence that SARS-CoV-2 infects neurons directly. SARS-CoV-2 might enter the brain via the olfactory bulb, where SARS-CoV-2 protein has been found in brain vascular endothelium [116]. There is little evidence for the presence of SARS-CoV-2 viral RNA in the cerebrospinal fluid of living patients [117,118]. The low detection rates of SARS-CoV-2 viral RNA and proteins in the brain tissue of patients who died acutely from COVID-19 suggest possible viral contamination during autopsy or detection of haematogenous viral RNA [100,102,103,116,119,120]. Moreover, there is no difference in detection rate of the virus in brain tissue between patients with and without neurological symptoms [103]. These findings indicate that viral invasion of the brain is unlikely to be responsible for long-term symptoms.

### Psychological impacts

Psychological responses to illness are well-studied phenomena, although there is great variation in the way different people deploy strategies to cope with the stress of illness [121]. People who are depressed or anxious can show evidence of cognitive impairment [122]. A large international survey of factors associated with psychiatric outcomes compared PCC patients (*n* = 5638) to individuals who had recovered from COVID-related symptoms (*n* = 475) [123]. Although PCC patients had a higher mental health burden, the majority (57%) did not report experiencing depression, anxiety, or suicidality. Indeed, there was also no evidence in this study of maladaptive coping in the PCC group. Nevertheless, it is also clear, from this and the reports discussed in sections above, that many people with PCC have mental health symptoms which are also associated with impaired cognition, although the direction of causality remains to be established.

## Concluding remarks

COVID-19 infection can have a negative impact on brain health and cognition in some individuals, both acutely and months after infection. We have presented data from a range of studies using electronic health records, community-based surveys, cognitive assessments, and neuroimaging that reveal both the complexity and limitations of current knowledge in this area. The known risks of significant acute and chronic effects on mental health and cognitive function were considered. Crucially, these impacts are not only limited to people who had severe symptoms of acute illness or those who experienced long COVID, and can also be evident in milder cases without ongoing symptoms.

PCC continues to be a global public health concern. Persistent cognitive symptoms are common after a SARS-CoV-2 infection. However, there are still many important questions regarding the long-term cognitive effects of COVID-19 that have not been resolved (see Outstanding questions). Understanding the underlying mechanisms is still in its infancy. It will rely on high-quality studies that integrate both self-reported symptoms and objective cognitive function testing, as well as specific variables such as virus variants, vaccination status, and pre-existing conditions. Answering these questions will be essential to help clinicians and policymakers design interventions to treat and possibly treat long-term effects on cognition and brain health.

Outstanding questions Which cognitive deficits contribute to the symptom of 'brain fog'?

Which risk variables contribute to the frequency and degree of the post-COVID chronic cognitive deficit at an individual level? There is a pressing need for large-sample, interdisciplinary investigations.

Do the affected cognitive functions after COVID-19 all recover eventually? How is the recovery duration of mental health symptoms and cognitive deficits affected by risk variables? Which domains of cognition recover slowest?

How do the different impacts of COVID-19 interact with each other? For example, is the chronic cognitive deficit associated with neuropsychiatric vulnerability?

Are post-COVID cognitive deficits specific to particular viral variants?

How many different subtypes of cognitive problems (e.g., different clusters) are there, and how do they differ in terms of underlying mechanisms?

What is the natural evolution of abnormalities seen at brain imaging, and what is their long-term impact on cognitive function?

Alt-text: Outstanding questions
